# Influence of plant reproductive systems on the evolution of hummingbird pollination

**DOI:** 10.1002/ece3.8621

**Published:** 2022-02-17

**Authors:** Stefan Abrahamczyk, Maximilian Weigend, Katrin Becker, Lea Sophie Dannenberg, Judith Eberz, Nayara Atella‐Hödtke, Bastian Steudel

**Affiliations:** ^1^ 9374 Nees Institute for Biodiversity of Plants University of Bonn Bonn Germany; ^2^ 122238 Health and Environmental Sciences Xi'an Jiaotong‐Liverpool University Suzhou China

**Keywords:** bee, germination rate, outcrossing, pollination efficiency, seed set, selfing

## Abstract

Many hummingbird‐pollinated plant species evolved from bee‐pollinated ancestors independently in many different habitats in North and South America. The mechanisms leading to these transitions are not completely understood. We conducted pollination and germination experiments and analyzed additional reproductive traits in three sister species pairs of which one species is bee‐ and the other hummingbird‐pollinated. All hummingbird‐pollinated species showed higher seed set and germination rates in cross‐pollinated than in self‐pollinated flowers. In the self‐compatible, bee‐pollinated sister species this difference did not exist. As expected, seed set and germination rate were higher after cross‐pollination in the largely self‐incompatible genus *Penstemon* independently of the pollination syndrome. However, the bird‐pollinated species produce only half of the amount of ovules and pollen grains per flower compared to the bee‐pollinated sister species. This indicates that hummingbird pollination is much more efficient in self‐incompatible populations because hummingbirds waste less pollen and provide higher outcrossing rates. Therefore, hummingbird pollination is less resource costly. Overall, we suggest that hummingbirds may increase the reproductive success compared to bees, influencing the evolution of hummingbird pollination in ecosystems with diverse bee assemblages.

## Graphical abstract

We conducted pollination and germination experiments in three sister species pairs of which one species is bee‐ and the other hummingbird‐pollinated. It turned out that outcrossing—mostly provided by hummingbirds—strongly increased seed set, making hummingbird the more efficient pollinators compared to bees.

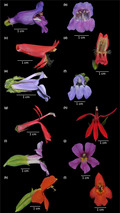

## 1 INTRODUCTION

Plant–animal and especially plant–pollinator interactions are known to be an important driver of plant biodiversity (Bascompte & Jordano, 2007; van der Niet & Johnson, 2012). Many plant species evolved because a population of one species became genetically isolated due to a switch to a new species or even group of pollinators (Kessler et al., 2020; van der Niet & Johnson, 2012). Sharp pollination isolation is obvious if this switch is between very different pollinators, for example, from insect to bird or from moth to bat, which commonly occurs. As an example, the switch from bee to hummingbird pollination has been documented for at least 70 independent plant lineages in North America only (Abrahamczyk & Renner, 2015). However, the evolutionary mechanisms leading to these switches are not fully understood.

In the entire Americas, hummingbird pollination has been documented for thousands of plant species from at least 404 genera and 68 families (Abrahamczyk & Kessler, 2015). The vast majority of hummingbird‐pollinated plant species as well as their pollinators inhabit the tropical cloud forests and adjacent (sub‐)alpine openland habitats (Abrahamczyk & Kessler, 2015; Krauss et al., 2017). At high elevations, especially in tropical cloud forests, hummingbirds are more effective pollinators than bees because they are less hindered by humid conditions and low temperatures (Cruden, 1972; Dellinger et al., 2021). This phenomenon has been widely used as an argument for the independent evolution of switches from bee to hummingbird pollination in these habitats. However, a considerable proportion of hummingbird‐pollinated plant lineages and species do not occur in humid and cool tropical cloud forests but in warmer and dryer, low‐ to mid‐elevations (e.g., Abrahamczyk & Renner, 2015). Hummingbird pollination is widely reported from tropical humid to arid openland and forest types well into the temperate zones of the Americas, where bees represent an abundant, diverse and efficient pollinator group (Freitas et al., 2009; Wilson & Carril, 2015). Therefore, the ecophysiological differences between the pollinator guilds do not provide a convincing explanation for the shift in pollination system in these habitats. An additional explanation is required for the multiple evolutions of hummingbird pollination (Thomson & Wilson, 2008).

A number of studies suggested that differences in bee and bird behavior are an important reason why bird pollination evolved numerous times from bee‐pollinated ancestors (Abrahamczyk, 2019; Kessler et al., 2020; Krauss et al., 2017). These studies argued that non‐territorial birds move much more between plant individuals and fly longer distances than bees. Thus, they get in contact with a larger number of plant individuals. Additionally, birds groom much less than bees and do not feed on pollen (Castellanos et al., 2003). Therefore, much less pollen is lost in transit, leading to higher pollen deposition rates (Castellanos et al., 2003; Mackin et al., 2021). The higher mobility, larger ranges (>1 km radius around range center) and higher pollen deposition rates of birds compared to bees are expected to increase outcrossing rates in bird‐pollinated plants (Abrahamczyk, 2019; Krauss et al., 2017). In contrast, most bees commonly have ranges with a radius of a few hundred meters and bee pollination involves higher pollen losses due to grooming and pollen consumption, leading to higher selfing rates (Karron et al., 2009; Krauss et al., 2017).

In addition, it seems plausible that not only the discrepancy in the behavior of the different groups of pollinators but also the reproductive system of the involved plant populations influence the evolution of hummingbird pollination. But comparative experimental analyses of reproductive success over a large set of hummingbird‐pollinated species evaluating the influence of the reproductive system are scarce, even though important. Only one study (Wolowski et al., 2013) compared fruit sets induced by self‐ and cross‐pollinations in 78 species from the Atlantic Rainforests. It found that most species were able to self‐pollinate, nevertheless, in most species fruit set was at least slightly higher if flowers were cross‐pollinated. But, fruit set is not a very informative trait to measure reproductive success, especially in species with multi‐seeded fruits. It just provides information on the presence of fruits and not on the proportion of developed seeds. For example, in largely self‐incompatible species ovules fertilized by own pollen often stop developing and produce sterile seeds or seeds with a reduced viability (Bittencourt & Semir, 2004; Duarte et al., 2017; Gibbs, 2014). However, several single species studies (Bertin, 1982; Schemske, 1980; Waser & Price, 1989) indicate that cross‐pollination increases seed‐set in hummingbird‐pollinated species, underlining the impact of plant reproductive systems on the evolution of these species.

In contrast to single species studies, sister species analyses comparing reproductive success of bee‐ and bird‐pollinated species are a more powerful tool to disentangle the impact of plant reproductive systems on the evolution of hummingbird pollination. If hummingbirds start visiting a number of bee‐pollinated plant populations that vary in their degree of self‐compatibility, visitation by hummingbirds will probably influence seed set in the individual species differently. The increase in seed set induced by hummingbirds is likely highest in those populations that are most dependent on outcrossing because due to their larger ranges and higher pollen deposition rates hummingbirds are more effective outcrossers than bees. Thus, one may assume that the evolution of hummingbird pollination is most likely in plant populations that depend on cross‐pollination and that hummingbird‐pollinated species are more dependent on outcrossing than their bee‐pollinated sister species.

Independent on the degree of self‐incompatibility of the individual species, hummingbird pollination might evolve because it is less costly. Pollination by hummingbirds is less affected by pollen loss through grooming and consumption, increases the level of outcrossing and reduces the amount of own pollen on the stigma and potentially abortive selfed ovules. Therefore, a switch to hummingbird pollination may permit a reduction of pollen production, since less pollen is required for pollination, leading to increased resource (nitrogen as a major component in pollen) efficiency. Numerous hummingbird‐pollinated plant species even evolved anti‐bee mechanisms hindering bees to enter the flower, consume pollen, and possibly self‐pollinate (Clark et al., 2015; Coimbra et al., 2020; Wilson et al., 2006), which may be interpreted as a support of our hypotheses.

To test our assumptions, we conducted pollination experiments for three pairs of sister species. Each pair consists of one bee‐pollinated and one hummingbird‐pollinated species. All species pairs go back to bee‐pollinated ancestors. We analyzed seed set, seed weight, and germination rates. Additionally, we provide pollen and ovule numbers. We hypothesize that:

1. Selfing reduces seed set and germination rate in hummingbird‐pollinated species.
2. Selfing does not affect seed set or germination rates in self‐compatible, bee‐pollinated species.
3. Hummingbird pollination reduces the reproduction costs in self‐incompatible species compared to bee pollination, that is, the number of pollen grains that need to be produced for successful reproduction.

## 2 MATERIAL AND METHODS

### 2.1 Plant material

**FIGURE 1:**
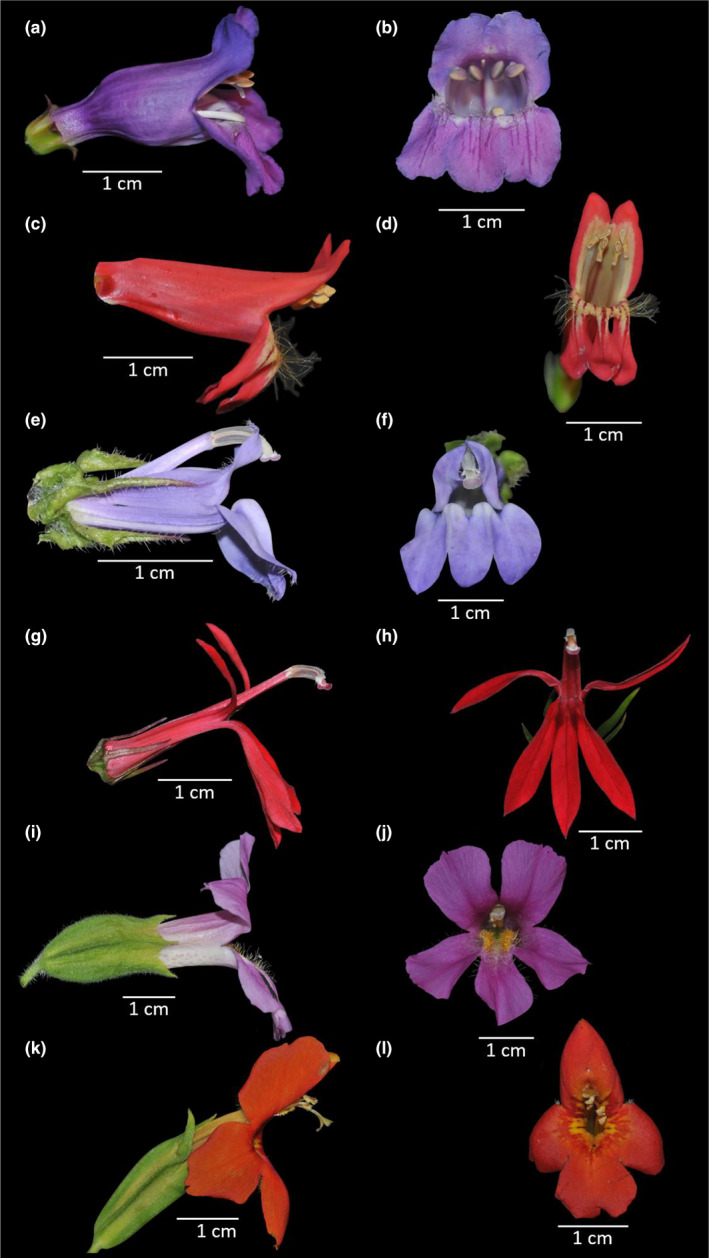
The three investigated sister species pairs, on the left in the lateral view and on the right in the frontal view. The first mentioned species of each genus is bee‐pollinated and the second one hummingbird‐pollinated. (a, b) *Penstemon neomexicanus*; (c, d) *Penstemon barbatus*; (e, f) *Lobelia siphilitica*; (g, h) *Lobelia cardinalis*; (i, j) *Mimulus lewisii*; (k, l) *Mimulus cardinalis*

For our study we used three sister species pairs of bee‐ and hummingbird‐pollinated plants from three genera corresponding to three different plant families (Figure 1). The most recent common ancestor of each species pair was bee‐pollinated. To cover the range of reproductive strategies in hummingbird‐pollinated plants we chose one fully self‐compatible species pair (*Mimulus cardinalis – Mimulus lewisii*, Phrymaceae; Schemske & Bradshaw, 1999; Beardsley et al., 2003), one partly self‐compatible pair (*Lobelia cardinalis – Lobelia siphilitica*, Campanulaceae; Johnston, 1992; Chen et al., 2016), and one largely self‐incompatible pair (*Penstemon barbatus – Penstemon neomexicanus*, Plantaginaceae; Lange et al., 2000; Wessinger et al., 2019). All species are distributed in North America in a range of habitats, including wetlands, meadows, and dry open forest from lowlands to the forest line. Seeds of all species were obtained from natural populations (via Alplains, www.alplains.com or Botanical Gardens) and young plants were raised in the Botanical Gardens of Bonn University in Germany. For documentation we took herbarium specimens of all species and deposited them in the herbarium of Bonn University (voucher numbers: *Mimulus cardinalis* 2759, *M*. *lewisii* 2758, *Lobelia cardinalis* 2828, *L*. *siphilitica* 2827, *Penstmon barbatus* 2757, *P*. *neomexicanus* 2953).

### 2.2 Pollen grain and ovule counts

To determine the pollen grain production per flower we collected five pre‐anthetic flowers from five different plant individuals each (total 25 flowers/species). We transferred the closed anthers of each flower into individual Eppendorf tubes. These were left to dry at room temperature for at least 48 h with an open lid. Afterwards, we added 200 µl glycerol to each sample and mixed it for 5 min with a laboratory mixer mill at 200 Hz (Retsch MM 200; Retsch, Haan, Germany). Samples were then placed in an ultrasonic bath (Sonorex Rk 52; Bandelin, Berlin, Germany) for 15 min each. Finally, we vortexed each sample and transferred 20 µl of the unstained mixture into a hemocytometer containing a Fuchs–Rosenthal counting chamber with 16 squares. Pollen grains were then counted in five randomly chosen large squares under a microscope (Axio, Scope.A1, Zeiss) and the total number of pollen grains per flower was calculated.

To count the number of ovules we used the same 25 flowers as for counting pollen grains. Since the ovules of *Lobelia* and *Mimulus* are minute and numerous we cut the ovary in several parts and counted the ovules in aliquots under a stereo microscope and totaled up the counts. We quantified pollen grain number and ovule number of the same flower separately to calculate ovule/pollen ratio of the individual flowers.

### 2.3 Pollination experiments

All autogamy as well as the manual self‐ and cross‐pollination experiments were conducted in a pollinator‐proof greenhouse. We used five flowers each of five plant individuals (25 flowers) each per species and pollination treatment. Only 19 flowers for self‐pollination were available for *Lobelia cardinalis*. Additionally, one individual of *Penstemon barbatus* died during the experiment. Thus, we used only four individuals of this species in the individual experiments, but increased the number of harvested fruits per plant. Thus, in the end we also used 25 fruits per treatment of *Penstemon barbatus*. For the autogamy treatment we just marked the flowers and left them un‐manipulated. For each single pollination of the manual self‐ and cross‐pollination experiments, we used pollen from two flowers in both treatments. We took freshly opened anthers with a pair of pincers and dusted the stigma until it was covered with pollen. Treatments were coded on the pedicels with differently colored cotton yarn. Instruments used were cleaned with alcohol in between each pollination experiment.

### 2.4 Seed counts and seed weight

We harvested all capsules when the sutures started to open and let them dry completely in paper bags at room temperature. After drying, we opened each fruit, transferred the seeds into a petri dish and counted them under a stereo microscope. Only fully developed seeds were included in the counts.

Seed weights were determined only for the seeds resulting from the manual self‐ and cross‐pollination experiments with an analytic balance (Mettler‐Toledo, XS205 Dual Range). The species of *Lobelia* and *Mimulus* have very small and light seeds. Therefore, we counted out 10 times 10 individual seeds per capsule together and calculated a mean seed weight per capsule. The seeds of the *Penstemon* species are much larger and much less numerous per capsule. Thus, we weighed all fully developed seeds per capsule individually and calculated a mean per capsule.

### 2.5 Germination experiments

For the germination experiment we applied only seeds resulting from the manual self‐ and cross‐pollination experiments. We used 100 seeds per capsule for the *Lobelia* and *Mimulus* species and all seeds from the *Penstemon* species. The seeds of *Lobelia* and *Mimulus* require no stratification and were stored for 8 weeks in paper bags in a fridge at 5°C prior to the germination experiments. *Penstemon* seeds require moisture stratification (Meyer & Kitchen, 1994) and were therefore stored on moist filter paper in closed petri dishes for 8 weeks in a fridge at 5°C. Seeds were surface sterilized before sown by placing them into Eppendorf tubes with one drop of polysorbat (Tween 20) and 1.5 ml of 3% sodium hypochlorite solution. After incubation at room temperature for 10 min we hydro‐extracted the mixture and took off the liquid. Afterwards, seeds were washed 10 times with 1.5 ml of sterilized water each. For germination, all seeds were placed on moist filter paper in closed petri dishes and kept in an incubator at 18°C for 2 weeks. Evaporated water was replaced three times per week with autoclaved water. Germination rates were determined after 2 weeks when most viable seeds have been germinated.

### 2.6 Statistics

In order to make seed set comparable across taxa and treatments we divided seed set by mean ovule number per species. We called the resulting variable relative seed set. We only analyzed data resulting from the manual self‐ and cross‐pollination experiments since the autogamy experiments let to too few fruits.

We applied linear regression models to test whether relative seed set, seed weight, or germination rate are significantly influenced by pollinator group or pollination treatment (only the manual self‐ and cross‐pollination experiments) using the “lm“ function in R 4.0.2 (R Core Team, 2020). To evaluate the interaction between the genera, we calculated two linear models for all traits, one without interaction and one with interaction and compared them using the “anova“ function in R. If no significant difference between the tested models was revealed, we chose the model without interaction in congruence with simplicity.

Additionally, we conducted linear regression models to find out whether the number of ovules and pollen grains per flower significantly differ between sister species with different pollinator groups.

To evaluate whether hummingbird‐pollinated species benefit from outcrossing, we conducted *t*‐tests for the individual species using the command “t.test” to explore differences between the experimental pollination treatments for relative seed set, seed weight, and germination rate. To observe changes of ovule and pollen grain number as well as for the ratio of both we conducted t‐tests between the hummingbird‐ and bee‐pollinated sister species within each genus accordingly.

## 3 RESULTS

**FIGURE 2:**
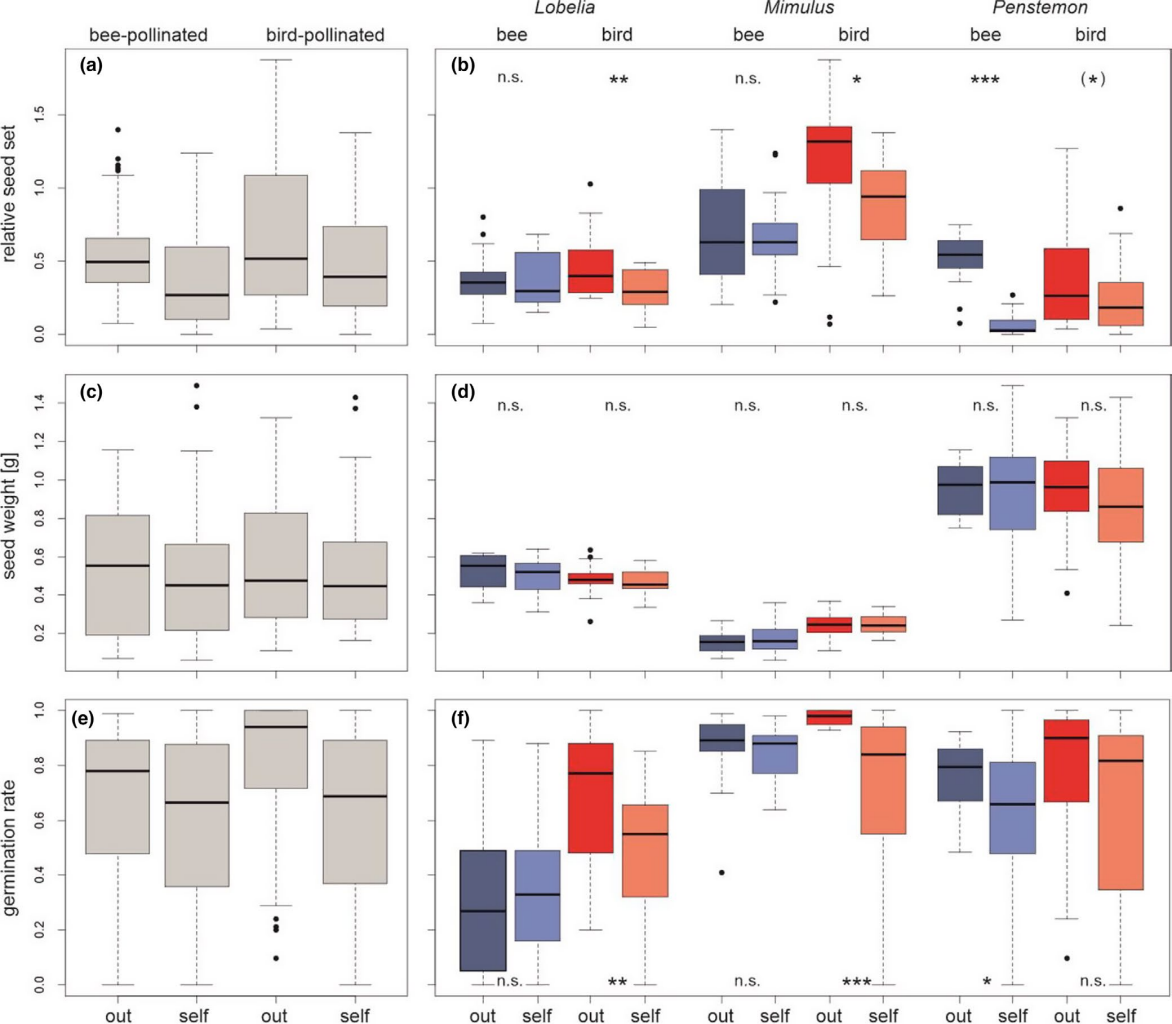
Differences between relative seed sets (a, b), seed weights (c, d), and germination rates (e, f) of hummingbird‐ and bee‐pollinated sister species pairs under self‐ and cross‐pollinations. The boxplots on the left side (a, c and e) show the groups used for the linear models while parts b, d and f show the data divided into species and pollination group with the results of the *t*‐tests between the outcrossing and the selfing treatment for the single species. Significance was indicated as ***p ≤ .001, **p ≤ .01, *p ≤ .05, ^(^*^)^ *p* ≤ .1, and n.s. = not significant. For the genera, “bee” indicates the bee‐pollinated species (blue colors), while “bird” indicates the hummingbird‐pollinated species (red colors). For the treatment, “out” represents the outcrossed group (darker colors), while “self” represents the self‐pollinated (brighter colors) group

Relative seed set and germination rate were influenced by both pollinator group and pollination treatment, but seed weight was not affected (Figure 2, Table 1). In self‐compatible hummingbird‐pollinated species (*Lobelia* and *Mimulus*) relative seed set as well as germination rate were reduced if the flowers were self‐pollinated in comparison to cross‐pollination. For the bee‐pollinated species of these genera, no difference of relative seed set and germination rate was found, indicating that the evolution of hummingbird pollination was combined with an increase in self‐incompatibility in these genera. In contrast, in the self‐incompatible genus *Penstemon*, the relative seed set of the hummingbird‐pollinated species was only marginally (*p* = .07) increased in the cross‐ compared to the self‐pollination treatment, while germination rate did not show differences between self‐ and cross‐pollination. But in the bee‐pollinated species cross‐pollination led to a higher relative seed set and germination rate than self‐pollination. The results of the largely self‐incompatible genus *Penstemon* are thus in contrast to the results of the largely self‐compatible genera *Lobelia* and *Mimulus*. The autogamy experiment did not result into fruit or seed production in *Lobelia* and *Mimulus*. Only in *Penstemon neomexicanus* seven fruits with a mean of 2.71 seeds were produced while in *Penstemon barbatus* five fruits with a mean of 1.2 seeds developed. Thus, all investigated species can be regarded as pollinator‐dependent.

**TABLE 1:** Results of the linear regression models for relative seed set, seed weight, and germination rate

	Estimate	SE	*T*‐value	*p*‐value
Relative seed set (SAM)				
Bee‐pollinated outcrossed	0.523	0.037	14.16	<.001
Difference of hummingbird pollination	0.128	0.043	2.95	.003
Difference of selfing	−0.171	0.043	−3.92	<.001
Seed weight				
Bee‐pollinated outcrossed	0.546	0.034	16.27	<.001
Difference of hummingbird pollination	0.013	0.039	0.32	.75
Difference of selfing	−0.033	0.039	−0.83	.41
Germination rate				
Bee‐pollinated outcrossed	0.688	0.03	23.23	<.001
Difference of hummingbird pollination	0.102	0.035	2.95	.003
Difference of selfing	−0.132	0.035	−3.81	<.001

Note

That the estimates are given for the first line (bee‐pollinated outcrossed) in comparison with zero and the following in comparison with first line results for all models, respectively.

**FIGURE 3:**
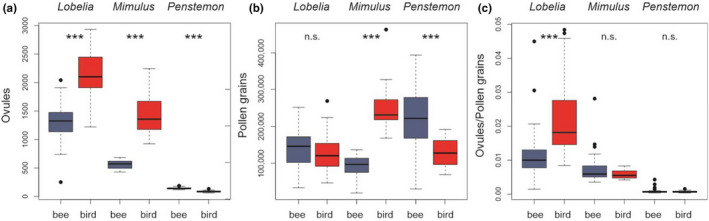
Boxplots of (a) ovule numbers, (b) pollen grain numbers and (c) the ratio of ovules and pollen per flower. Differences between hummingbird‐ (red) and bee‐pollinated (blue) sister species are tested by t‐tests. Significance was indicated as *** *p* ≤ .001 and n.s. = not significant. On the x‐axes, “bee” indicates the bee‐pollinated species, while “bird” indicates the hummingbird‐pollinated species

In the three sister species pairs the production of ovules and pollen was highly variable: In *Lobelia* and *Mimulus* ovule numbers were higher in the hummingbird‐pollinated than in the bee‐pollinated sister species, while in *Penstemon* it was the opposite (Figure 3a, Table 2). Pollen grain production strongly varied within and between sister species pairs (Figure 3b, Table 2): In *Lobelia*, pollen grain number did not differ between sister species. In *Mimulus*, the hummingbird‐pollinated species produced more pollen grains than the bee‐pollinated species. Finally, in *Penstemon* the bee‐pollinated species produced more pollen grains than the hummingbird‐pollinated sister species. Thus, ovule/pollen ratio was significantly higher in the hummingbird‐pollinated *Lobelia cardinalis* in comparison to *Lobelia siphilitica*, indicating less pollen grains produced per ovule in *Lobelia cardinalis*. The ratios in the other two sister species pairs did not differ significantly (Figure 3c, Table 2).

**TABLE 2:** Results of the linear regression models for ovule and pollen grain number and the ratio of both

	Estimate	SE	*T*‐value	*p*‐value
Pollen				
Bee‐pollinated *Lobelia*	137496	11552	11.90	<.001
Difference of bird‐pollinated *Lobelia*	−1646	16338	−1.01	.315
Difference of bee‐pollinated *Mimulus*	−49064	16338	−3.00	.003
Difference of bee‐pollinated *Penstemon*	76800	16338	4.70	<.001
Difference of bird‐pollinated *Mimulus*	176744	23105	7.65	<.001
Difference of bird‐pollinated *Penstemon*	−69280	23105	−3.00	.003
Ovules				
Bee‐pollinated *Lobelia*	1289.6	52.97	24.35	<.001
Difference of bird‐pollinated *Lobelia*	884.6	74.91	11.81	<.001
Difference of bee‐pollinated *Mimulus*	−728.6	74.91	−9.73	<.001
Difference of bee‐pollinated *Penstemon*	−1143.8	74.91	−15.27	<.001
Difference of bird‐pollinated *Mimulus*	−19.1	105.94	−0.18	.857
Difference of bird‐pollinated *Penstemon*	−943.0	105.94	−8.90	<.001
Ovules/pollen				
Bee‐pollinated *Lobelia*	0.012	0.001	8.98	<.001
Difference of bird‐pollinated *Lobelia*	0.010	0.002	5.51	<.001
Difference of bee‐pollinated *Mimulus*	−0.004	0.002	−2.24	.003
Difference of bee‐pollinated *Penstemon*	−0.011	0.002	−5.84	<.001
Difference of bird‐pollinated *Mimulus*	−0.012	0.003	−4.64	<.001
Difference of bird‐pollinated *Penstemon*	−0.011	0.003	−3.98	<.001

Note

That the estimates are given for the first line (bee‐pollinated *Lobelia*) in comparison with zero and the following in comparison with first line results for all models respectively.

## 4 DISCUSSION

In congruence with our hypotheses, we found that reproductive systems may influence the evolution of hummingbird pollination in three sister species pairs. Plant populations from regions with abundant and diverse bee assemblages probably adapted to hummingbird pollination because reproductive success of these populations is promoted by higher outcrossing rates provided by hummingbirds compared to bees (Castellanos et al., 2003; Krauss et al., 2017). We found that in all hummingbird‐pollinated species—independent of their selfing ability—selfing reduced seed set, but in the self‐incompatible genus *Penstemon* this trend was only marginally significant. Additionally, germination rate was reduced by selfing in hummingbird‐pollinated *Lobelia* and *Mimulus* species. In contrast, in the bee‐pollinated species we found significant differences only in *Penstemon neomexicanus*. The self‐compatible, bee‐pollinated sister species in *Lobelia* and *Mimulus* did not show significant differences in seed set or germination rates between self‐pollination and outcrossing. The confirmation of the outcrossing nature of hummingbird‐pollinated species largely supports our hypotheses and underlines that the reproductive system may play an important role in the evolution of hummingbird pollination systems.

For largely self‐incompatible genera with bee‐ and hummingbird‐pollinated species, such as *Penstemon* (Clements et al., 1999; Lange et al., 2000; Lange & Scott, 1999; Wolfe et al., 2014) differences in seed set and germination rate between self‐ und cross‐pollination treatments are commonly expected independent of the pollen vector. However, independent of the plant´s reproductive system bees often deposit more own pollen on the stigmas than hummingbirds, enabling a higher degree of selfing (Krauss et al., 2017). This behavior‐induced difference of the two pollinator groups may have led to the evolution of stronger selfing barriers in bee‐pollinated than in hummingbird‐pollinated *Penstemon* species (Lange et al., 2000; Lange & Scott, 1999; Tepedino et al., 2007; Zorn‐Arnold & Howe, 2007). Finally, the high probability of seed abortions in self‐incompatible bee‐pollinated plant lineages may force the evolution of hummingbird pollination in these lineages.

Additionally, in self‐incompatible lineages a switch from bee to hummingbird pollination may also be more economical in terms of resources because hummingbirds waste less pollen and facilitate cross‐pollination. Thus, less ovules are fertilized by own pollen leading to less abortions and less sterile or less viable seed development (Bittencourt & Semir, 2004; Duarte et al., 2017). In line with this argument, the hummingbird‐pollinated *Penstemon barbatus* produces only about half of the amount of pollen grains and ovules compared to its bee‐pollinated sister species *Penstemon neomexicanus*. Due to the higher outcrossing rate provided by hummingbirds compared to bees (Krauss et al., 2017) it seems very likely that a higher proportion of ovules develops into seeds in *Penstemon barbatus* than in *Penstemon neomexicanus*. But comparative pollination studies in natural populations of both *Penstemon* species would be necessary to prove this hypothesis. We interpret the lower number of ovules and pollen grains in *Penstemon barbatus* as reflecting a more parsimonious and more efficient pollination mechanism, leading to potentially fewer seeds, but a higher proportion of cross‐pollinated, viable seeds.

Whether such resource‐saving mechanisms also exist in self‐compatible, hummingbird‐pollinated species remains unclear. Indeed, we found that also the self‐compatible, hummingbird‐pollinated *Lobelia cardinalis* has a higher number of ovules than the bee‐pollinated *Lobelia spiphilitica*, but similar numbers of pollen grains, indicating a lower ovule/pollen ratio, which in turn indicates higher pollination efficiency. This is a different mechanism—reducing the pollen production relative to ovule production—but likely with the same overall effect as in *Penstemon*, that is, the reduction of resource investment in gametes relative to the seed number produced. In *Penstemon*, however, the mechanism appears to be focused on seed quality (production of outcrossed seed) rather than overall seed production. However, we can only speculate why we find a parallel increase of pollen grains and ovules in *Mimulus cardinalis*, which goes against our resource saving hypothesis. One possible explanation may be that *Mimulus cardinalis* occur in naturally nutrient‐rich habitats, such as river banks (Ramsey et al., 2003) and may therefore not be nutrient‐limited.

Based on the comparison of three sister pairs of bee‐ versus hummingbird‐pollinated species, we argue that the reproductive system is an important factor in the evolution of hummingbird pollination. Increasing reproductive success associated with hummingbird pollination resulted into higher seed quality and maybe higher resource efficiency. We tentatively suggest that the increase in reproductive success in hummingbird pollination relative to bee pollination (higher outcrossing rates) may be one driving force behind the evolution of hummingbird pollination in ecosystems where bees are diverse and abundant. The mechanism described in this study may also be used to explain the evolution of other pollination systems, such as hawkmoth or bat pollination but experiments applying sister species pairs with different pollinator groups are necessary to prove this hypothesis. Clearly, a wider sampling across a range of sister groups and well‐resolved phylogenies would be required to confirm this conclusion. Field experiments involving the study of actual pollen transfer rates and especially from tropical lowland ecosystems would be particularly desirable to validate the results here obtained for temperate, herbaceous species.

## CONFLICT OF INTEREST

None declared.

## AUTHOR CONTRIBUTIONS

**Stefan Abrahamczyk:** Conceptualization (lead); Data curation (lead); Formal analysis (supporting); Investigation (lead); Supervision (lead); Writing – original draft (lead). **Maximilian Weigend:** Conceptualization (supporting); Investigation (supporting); Resources (lead); Supervision (supporting); Writing – original draft (supporting). **Katrin Becker:** Investigation (supporting). **Lea Sophie Dannenberg:** Investigation (supporting). **Judith Eberz:** Investigation (supporting). **Nayara Atella‐Hödtke:** Investigation (supporting). **Bastian Steudel:** Formal analysis (lead); Writing – original draft (supporting).

## Data Availability

All data are published at Dryad (https://doi.org/10.5061/dryad.bnzs7h4cj).
